# Structure Differentiation of Hydrophilic Brass Nanoparticles Using a Polyol Toolbox

**DOI:** 10.3389/fchem.2019.00817

**Published:** 2019-11-29

**Authors:** Orestis Antonoglou, Evangelia Founta, Vasilis Karagkounis, Eleni Pavlidou, George Litsardakis, Stefanos Mourdikoudis, Nguyen Thi Kim Thanh, Catherine Dendrinou-Samara

**Affiliations:** ^1^Laboratory of Inorganic Chemistry, Department of Chemistry, Aristotle University of Thessaloniki, Thessaloniki, Greece; ^2^Department of Physics, Aristotle University of Thessaloniki, Thessaloniki, Greece; ^3^Laboratory of Materials for Electrotechnics, Department of Electrical and Computer Engineering, Aristotle University of Thessaloniki, Thessaloniki, Greece; ^4^Biophysics Group, Department of Physics and Astronomy, University College London (UCL), London, United Kingdom; ^5^UCL Healthcare Biomagnetic and Nanomaterials Laboratories, London, United Kingdom

**Keywords:** bimetallic nanoparticles, copper, zinc, triethylene glycol, polyol process, microwave, solvothermal

## Abstract

Nano-brasses are emerging as a new class of composition-dependent applicable materials. It remains a challenge to synthesize hydrophilic brass nanoparticles (NPs) and further exploit them for promising bio-applications. Based on red/ox potential of polyol and nitrate salts precursors, a series of hydrophilic brass formulations of different nanoarchitectures was prepared and characterized. Self-assembly synthesis was performed in the presence of triethylene glycol (TrEG) and nitrate precursors Cu(NO_3_)_2_·3H_2_O and Zn(NO_3_)_2_·6H_2_O in an autoclave system, at different temperatures, conventional or microwave-assisted heating, while a range of precursor ratios was investigated. NPs were thoroughly characterized via X-ray diffraction (XRD), thermogravimetric analysis (TGA), scanning electron microscopy (SEM), transmition electron microscopy (TEM), Fourier-transform infrared (FTIR) spectroscopy, dynamic light scattering (DLS), and ζ-potential to determine the crystal structure, composition, morphology, size, state of polyol coating, and aqueous colloidal stability. Distinct bimetallic α-brasses and γ-brasses, α-Cu_40_Zn_25_/γ-Cu_11_Zn_24_, α-Cu_63_Zn_37_, α-Cu_47_Zn_10_/γ-Cu_19_Zn_24_, and hierarchical core/shell structures, α-Cu_59_Zn_30_@(ZnO)_11_, Cu_35_Zn_16_@(ZnO)_49_, α-Cu_37_Zn_18_@(ZnO)_45_, Cu@Zinc oxalate, were produced by each synthetic protocol as stoichiometric, copper-rich, and/or zinc-rich nanomaterials. TEM sizes were estimated at 20–40 nm for pure bimetallic particles and at 45–70 nm for hierarchical core/shell structures. Crystallite sizes for the bimetallic nanocrystals were found ca. 30–45 nm, while in the case of the core-shell structures, smaller values around 15–20 nm were calculated for the ZnO shells. Oxidation and/or fragmentation of TrEG was unveiled and attributed to the different fabrication routes and formation mechanisms. All NPs were hydrophilic with 20–30% w/w of polyol coating, non-ionic colloidal stabilization (−5 mV < ζ-potential < −13 mV) and relatively small hydrodynamic sizes (<250 nm). The polyol toolbox proved effective in tailoring the structure and composition of hydrophilic brass NPs while keeping the crystallite and hydrodynamic sizes fixed.

## Introduction

Bimetallic nanoparticles (BMNPs) have gained excessive interest over the last few years, as they appear to be ideal candidates for a wide range of applications, such as catalysis, agrochemistry, optoelectronics, and biomedicine (Wang et al., 2010; Gilroy et al., 2016; Nasrabadi et al., 2016; Srinoi et al., 2018). The attraction for BMNPs can be attributed to unique characteristics of synergistic action of the two distinct elements that often gives rise to collective properties (Song et al., 2009; Perdikaki et al., 2016; Antonoglou et al., 2017). Among them, the substitutional bulk alloy of the bioessential metals copper and zinc known as brass is dated to be used centuries ago because of the superior mechanical, anticorrosion, and germicidal properties (Kharakwal and Gurjar, 2006; Mehtar et al., 2008; Michels et al., 2008). The classification of brass alloys can be established according to the proportion of copper and zinc contained, where different characteristics also emerge (Wiame et al., 2008; Hong et al., 2014; Keast et al., 2015; Liu and Cheng, 2019). Copper-rich phases that crystallize in fcc structure, known as α-brasses, are considered to be the most stable, containing a maximum of 38% Zn. Duplex brass, or αβ-brass, is characterized by limited ductility, with a maximum of 42% Zn content, where β-brass with a minimum of 45% Zn is the intermetallic form of this alloy, displaying a bcc crystal structure. Finally, the zinc-rich γ-phase of brass is described by a 57–68% Zn content and an hcp crystal structure. This phase is rather unstable, rendering it as quite challenging to be obtained via synthetic pathways (Gourdon et al., 2007). Apart from pure bimetallic phases, the heterostructure of Cu and ZnO is also considered a brass material (Qi et al., 2009).

Nowadays, a growing interest concerns nanoscale brass where additional advantages owing to nano-size can be exploited (Shishidoa et al., 2007; Qi et al., 2009; Minal and Prakash, 2016; Manna et al., 2017; Gentzen et al., 2018; Jiang et al., 2019). A variety of synthetic routes have been reported for nanobrass, mainly top-down methods, such as laser ablation or ball milling, while wet chemistry approaches are scarcely reported. Specifically, solvated metal atom dispersion (SMAD) method has been used for dealloying bulk brass (Bhaskar and Jagirdar, 2017). Brass NPs were generated by ablation of bulk brass in ethanol (Sukhova et al., 2014). Organometallic compounds such as [Cu{OCH(Me)CH_2_NMe_2_}_2_] and Et_2_Zn have been chosen as precursors in the synthesis of CuZn nanocolloids via their thermolysis in hot coordinating solvents (Hambrock et al., 2013). Another non-aqueous organometallic synthesis was performed by co-hydrogenolysis of [CpCu(PMe_3_)] and [${\text{ZnCp}}_{2}^{*}$] (Cokoja et al., 2006). Recently, brass alloy NPs were prepared under microwave-induced decomposition of the amidinate precursors {[Me(C(NiPr)_2_)]Cu}_2_ and [Me(C(NiPr)_2_)]_2_Zn in ([BMIm][BF_4_]) or in propylene carbonate (PC) liquids (Schütte et al., 2014). Moreover, the synthesis of Cu@ZnO core-shell NPs through digestive ripening process in SMAD, hot injection experiments by the decomposition of copper(I) chloride and zinc acetate in oleylamine, and surface modification with citric acid and ammonia as a precipitator have been reported (Yang et al., 2006; Kalidindi and Jagirdar, 2008; Chang et al., 2016). Meanwhile, various methods like sol-gel, electrodeposition, or procedures that include the use of plant extracts for the synthesis of different morphologies, have been investigated (Jamali-Sheini et al., 2014; Minal and Prakash, 2016; Manna et al., 2017; Jiang et al., 2019). However, in the majority of the methods referred above, the fabricated NPs were not hydrophilic, and this greatly limits some important applications as antimicrobial, agrochemical, and biomedical agents.

Among the plethora of wet chemical techniques, the polyol method seems to be quite advantageous in the synthesis of metal, metal oxides, and chalcogenides NPs (Altinçekiç and Boz, 2008; Fiévet et al., 2008; Dong et al., 2015a). In this process, polyalcohols are utilized in a triple role of solvent, stabilizing, and reducing agent, while their high boiling points and viscosity allow synthesis in relatively high temperatures, favoring the formation of well-crystallized products, without the need for post-annealing. The synthetic conditions enable an effective control over the physicochemical characteristics of the resulting products, specifically over size, shape, structure, surface chemistry (4Ss) and dispersibility contributing to the NPs' biocompatibility and hydrophilicity, along with the exclusion of by-product formation. Furthermore, most of the inorganic metal salt precursors are well-dissolved in these media, and organometallic precursors can be avoided. It has previously been reported in the literature (Caizer and Stefănescu, 2002; Stefănescu et al., 2007; Biacchi and Schaak, 2011; Carroll et al., 2011; Dong et al., 2015b; Teichert et al., 2018) and recently by us (Vamvakidis et al., 2015; Antonoglou et al., 2017, 2018; Giannousi et al., 2019; Tryfon et al., 2019) that synthetic conditions like polyol properties (structure, molecular weight, reducing ability, and capping capacity), reaction temperature, utilized precursors, and synthetic route affect the final structure and features of the nanostructures.

Recently, we reported a microwave-assisted polyol process (MW-PP) for the preparation of polyol coated nanobrass by using nitrate salt metal precursors and the biocompatible polyol triethylene glycol (TrEG) (Antonoglou et al., 2018). The applied process allowed us to isolate hydrophilic brass BMNPs where both the α-phase and γ-phase were formed with no zinc and/or copper oxide phases being produced. Such hydrophilic brass BMNPs displayed enhanced antifungal activity compared to monometallic copper NPs and no phytotoxicity, rendering them ideal candidates for agrochemical applications. Given that the 4Ss of BMNPs govern their activity, the present study gives rise to novel hydrophilic structures and compositions of brass BMNPs that can be crafted with the polyol toolbox. In that vein, a series of experiments are carried out where nitrate precursors Cu(NO_3_)_2_·3H_2_O and Zn(NO_3_)_2_·6H_2_O are mixed with TrEG, which acted in a triple role. A range of modifications in the reaction temperature, heating method, and precursor ratio conditions are investigated. Specifically, two reaction temperatures, 240 and 260°C, are evaluated while synthesis is carried out under either microwave-assisted or classic solvothermal conditions. Finally, precursor ratios of 2:1 and 1:2 of copper and zinc, respectively, are examined. All of the as-produced BMNPs are characterized in detail via X-ray diffraction (XRD), thermogravimetric analysis (TGA), scanning electron microscopy (SEM), transition electron microscopy (TEM), Fourier-transform infrared (FTIR) spectroscopy, dynamic light scattering (DLS), and ζ-potential to identify the crystal structure, composition, morphology, size, state of organic coating, and aqueous colloidal stability.

## Materials and Methods

### Microwave-Assisted Synthesis of Brass NPs

MW-PP was employed using a commercial microwave-accelerated reaction system, Model MARS 6-240/50-CEM. This system runs at a maximum frequency of 2,450 MHz and a power of 1,800 W. The reaction was carried out in a double-walled vessel consisting of an inner Teflon container liner where temperature and pressure sensors are connected and an outer composite sleeve. Zn(NO_3_)_2_·4H_2_O and Cu(NO_3_)_2_·3H_2_O were mixed and dissolved in 40 ml of TrEG, followed by transfer to an autoclave. After MW-PP, cooling of the autoclave till room temperature takes place during ~30 min, followed by centrifugation at 5,000 rpm, where supernatants were discarded, and a gray-black precipitate was acquired and washed three times with ethanol, for the removal of unreacted precursors.

Sample BM1: Zn(NO_3_)_2_·4H_2_O (2.0 mmol) and Cu(NO_3_)_2_·3H_2_O (2.0 mmol) were used. The reaction was carried out at 240°C with a hold time of 30 min and a ramp time heating step (from 25 to 240°C) set at 15 min.

Sample BM2: Zn(NO_3_)_2_·4H_2_O (2.0 mmol) and Cu(NO_3_)_2_·3H_2_O (2.0 mmol) were used. The reaction was carried out at 260°C with a hold time of 30 min and a ramp time heating step (from 25 to 260°C) set at 16 min.

Sample BM5: Zn(NO_3_)_2_·4H_2_O (1.33 mmol) and Cu(NO_3_)_2_·3H_2_O (2.67 mmol) were used. The reaction was carried out at 240°C with a hold time of 30 min and a ramp time heating step (from 25 to 240°C) set at 15 min.

Sample BM7: Zn(NO_3_)_2_·4H_2_O (2.67 mmol) and Cu(NO_3_)_2_·3H_2_O (1.33 mmol) were used. The reaction was carried out at 240°C with a hold time of 30 min and a ramp time heating step (from 25 to 240°C) set at 15 min.

### Solvothermal Synthesis of Brass NPs

A modified polyol process under solvothermal conditions in a Teflon container has been utilized. Zn(NO_3_)_2_·4H_2_O and Cu(NO_3_)_2_·3H_2_O were mixed and dissolved in 10 ml of TrEG, followed by the transfer to the autoclave. After the solvothermal process, the autoclave was cooled naturally to room temperature followed by centrifugation at 5,000 rpm, where supernatants were discarded, and a gray-black precipitate was acquired and washed three times with ethanol, for the removal of unreacted precursors.

Sample BM3: Zn(NO_3_)_2_·4H_2_O (0.5 mmol) and Cu(NO_3_)_2_·3H_2_O (0.5 mmol) were used. The reaction was carried out at 240°C with a hold time of 8 h and a ramp time heating step (from 25 to 240°C) set at 45 min.

Sample BM4: Zn(NO_3_)_2_·4H_2_O (0.33 mmol) and Cu(NO_3_)_2_·3H_2_O (0.67 mmol) were used. The reaction was carried out at 240°C with a hold time of 8 h and a ramp time heating step (from 25 to 240°C) set at 45 min.

Sample BM6: Zn(NO_3_)_2_·4H_2_O (0.67 mmol) and Cu(NO_3_)_2_·3H_2_O (0.33 mmol) were used. The reaction was carried out at 240°C with a hold time of 8 h and a ramp time heating step (from 25 to 240°C) set at 45 min.

All syntheses are given in Table 1.

**Table 1:** Syntheses of all samples.

**Sample**	**Heating method**	**Synthesis temperature (^**°**^C)**	**Ramp heating step (min)**	**Hold time**	**Zn(NO_**3**_)_**2**_ (mmol)**	**Cu(NO_**3**_)_**2**_ (mmol)**
BM1	Microwave	240	15	30 min	2	2
BM2	Microwave	260	16	30 min	2	2
BM3	Solvothermal	240	45	8 h	0.5	0.5
BM4	Solvothermal	240	45	8 h	0.33	0.67
BM5	Microwave	240	15	30 min	1.33	2.67
BM6	Solvothermal	240	45	8 h	0.67	0.33
BM7	Microwave	240	15	30 min	2.67	1.33

### Materials

All of the reagents were of analytical grade and were used without any further purification: Copper (II) nitrate trihydrate, Cu(NO_3_)_2_·3H_2_O (Merck, ≥99.5%, *M* = 241.60 g/mol), zinc (II) nitrate tetrahydrate, Zn(NO_3_)_2_·4H_2_O (Merck, ≥99.5%, *M* = 261.44 g/mol), and TrEG (Merck, ≥99%, *M* = 150.17 g/mol).

### Characterization of Brass NPs

The crystal structure of the synthesized NPs was investigated through XRD at ambient temperature on a Seifert XRD 3003-TT powder diffractometer using FeKa radiation in 2θ steps of 0.05° for 10 s and analyzed by the means of the Jade software (MDI's) to determine crystal structure and lattice parameters.

Powder morphology was determined by a JEOL 840A SEM coupled with energy dispersive X-ray spectrometer (EDX) for estimating the inorganic composition.

Particle size and morphology were determined by TEM. The images were acquired with a JEOL JEM 1200-EX microscope operating at an acceleration voltage of 120 kV. For TEM observations, suspensions of the NPs deposited onto carbon-coated copper grids were used.

FTIR (2,000–900 cm^−1^) was recorded using a Nicolet FT-IR 6700 spectrometer with samples prepared as KBr pellets.

TGA was employed using SETA-RAM SetSys-1200 and carried out in the range from room temperature to 900°C at a heating rate of 10°C min^−1^ under N_2_ atmosphere.

The hydrodynamic size and ζ-potential of brass NPs were determined by DLS measurements, carried out at 25°C utilizing a Nano ZS Malvern apparatus.

## Results and Discussion

### Synthetic Aspects

**Scheme 1:**
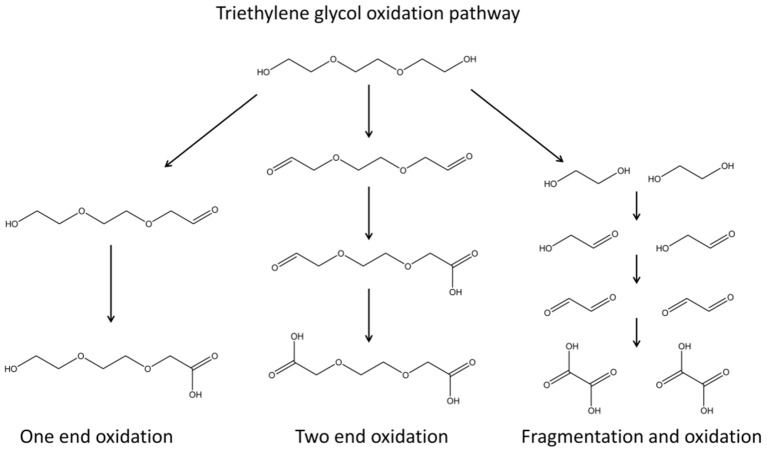
Triethylene glycol oxidation pathway; one-end and/or two-end oxidation as well as fragmentation paths can be followed.

Ligand-protected inorganic NPs are particularly attractive as interfacial effects impact the properties of both organic and inorganic components of these hybrid ensembles. The degree of NP aggregation can be controlled; terminal ligand groups determine the solubility of NPs and as such can be used to direct NP self-assembly. Taking into account the plethora of organic ligands that can be used, it is noted that a great number of possible nanosized materials can be generated by suitable combinations of organic and inorganic components. In this context, the polyol toolbox has been used to achieve an efficient wet chemical design for BMNPs formation, responsive to synthetic parameters, such as reaction temperature, conventional or microwave heating, and precursor ratio. Reduction and/or hydrolysis reactions can take place and favor the formation of metallic and/or oxide NPs, respectively (Poul et al., 2003). The fabrication mechanism follows the formation of polyol-metal complexes that decompose, giving rise to the nucleation and growth of brass BMNPs (Biacchi and Schaak, 2011; Carroll et al., 2011; Antonoglou et al., 2017, 2018; Teichert et al., 2018; Giannousi et al., 2019; Tryfon et al., 2019). Both the formation and decomposition of these intermediate polyol-metal complexes are very sensitive to synthetic regulations. Oxidation of TrEG occurs during the synthesis as seen in Scheme 1 where one-end and/or two-end oxidation as well as fragmentation paths can be followed (Caizer and Stefănescu, 2002; Stefănescu et al., 2007; Dong et al., 2015b; Vamvakidis et al., 2015; Antonoglou et al., 2017; Tryfon et al., 2019). As the heating method, ramp heating step, reaction temperature and hold time change, different oxidized forms and intermediates can be present, and finally different products are isolated. Additionally, precursor ratio and conventional or microwave heating affect the composition and structure of the products. Excess of either copper or zinc will favor the formation of copper-rich brasses or zinc-rich brasses/ZnO shells, respectively. Moreover, microwave-assisted polyol synthesis can even produce rather unstable bimetallic nanocrystals because of the rapid nucleation process, high temperature, and fast reaction time (Rao et al., 1999; Dahal et al., 2012). Hence, in the present study, each synthetic path tested gave rise to hierarchical structures and novel compositions of brass BMNPs as expected and analyzed below.

### Effect of Reaction Temperature

**Figure 1:**
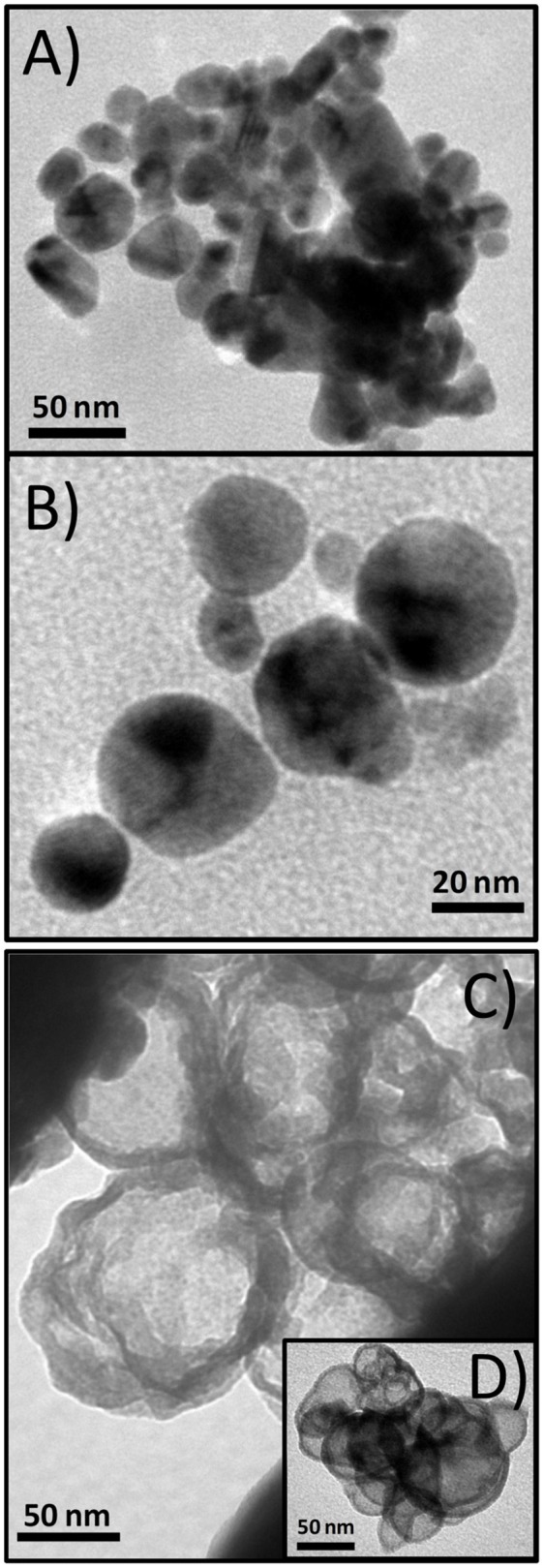
Transmition electron microscopy (TEM) captions of BM1, α-Cu_40_Zn_25_/γ-Cu_11_Zn_24_, **(A,B)**, and BM2, Cu@Zinc oxalate, bimetallic nanoparticles (BMNPs) **(C,D)**.

Temperature is a readily accessible factor for a number of different NP syntheses. To investigate the effect of the temperature, two samples, BM1 and BM2, were synthesized via the microwave-assisted process at 240 and 260°C, respectively, to get closer to TrEG's boiling point (285°C), where oxidation and fragmentation of the organic ligand can be promoted. The morphology and particle size of BM1 and BM2 were investigated with TEM images. Figure 1 presents snapshots for the BMNPs, where different morphologies are apparent. Truncated shapes with faceted corners were observed for BM1 (Figures 1A,B). In contrast, BM2 settled in a hierarchical type core/shell structure with more spherical cores and well-shaped shells (Figures 1C,D). Both BMNPs settle in nanoflower architectures that are not considered aggregates, as reported before (Gavilán et al., 2017). The particle sizes for BM1 and BM2 are estimated at 35–40 and 65–70 nm, respectively.

**Figure 2:**
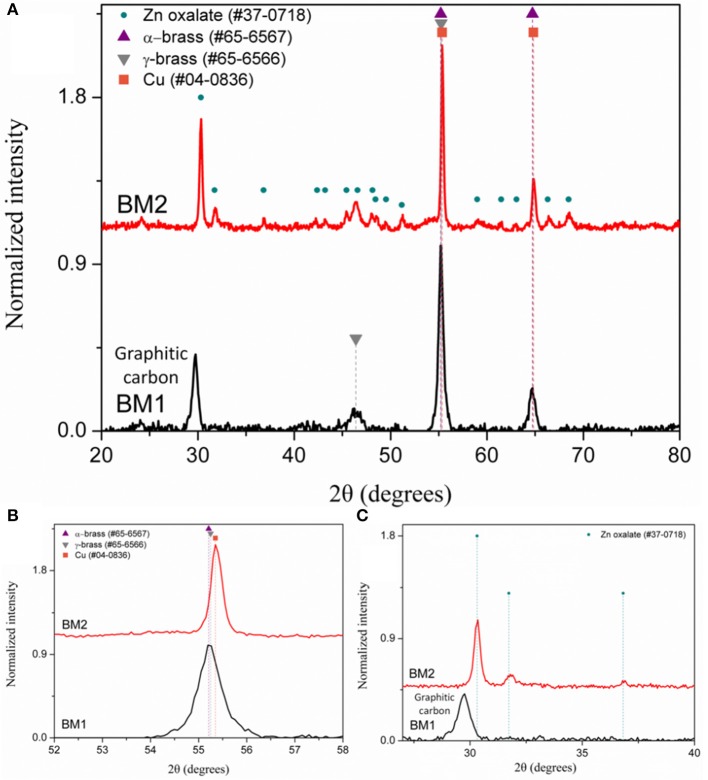
The X-ray diffraction (XRD) patterns of BM1 (synthesized at 240°C) and BM2 (synthesized at 260°C) at 20–80° **(A)**, 53–58° **(B)**, and 27–40° **(C)**, respectively.

The composition of BM1 and BM2 was estimated via SEM/EDX analysis (Figure S1) at Cu_50.8_Zn_49.2_ and Cu_49.7_Zn_50.3_, respectively, revealing that the ratio of precursors (1:1) was kept in the final composition. The crystal structure was investigated through XRD at room temperature (Figure 2). Both BM1 and BM2 displayed the peaks at around 55 and 65° that correspond to the fcc lattice adopted by α-brass (#65-6567, space group Fm-3m) and metallic copper (#04-0836, space group Fm-3m), whereas BM1 also contained the γ-brass phase (#65-6566, space group I-43m). Moreover, the black spots presented in the TEM image (Figures 1A,B) can be correlated to the α-brass and γ-brass phases that coexist in the BMNPs. However, it is clear from Figure 2B that BM1 crystallized as α-brass, whereas BM2 crystallized as metallic copper. This is verified by lattice parameter (a = b = c in the fcc lattice) values, estimated at 3.6159 Å (0.0028) and 3.6087 Å (0.0018) for BM1 and BM2, respectively, where the increase is attributed to the zinc doping of the fcc lattice. Additionally, in the case of BM2, Zn oxalate (#37-0718) was produced and is clearly distinct from the graphitic crystallite of BM1 (Figure 2C) (Dong et al., 2015b; Antonoglou et al., 2017, 2018; Tryfon et al., 2019). By utilizing the quantitative option of MDI's Jade, the % wt crystal composition is calculated at 65% α-brass and 35% γ-brass for BM1 and 50% Cu and 50% zinc oxalate for BM2. These results greatly emphasize the effect of reaction temperature in the MW-PP, where an increase of 20°C (BM2) led to the oxalate forms of TrEG-zinc intermediates. The stable zinc-oxalate tetrahedral complexes, with their stability being caused by chelate effect, failed to decompose and resulted in the 50% Cu and 50% zinc oxalate structure/composition (Giannousi et al., 2019). The crystallite sizes of BM2 were calculated by Scherrer equation at 45 nm for Cu and 30 nm for zinc oxalate and are in agreement with the 65–70 nm TEM size. The slightly bigger crystallite size is attributed to the biphasic state of BM2, where crystallites share volume in the particles and TEM size is smaller than the sum of the two crystallites. This supports the core-shell morphology revealed by TEM captions (Figures 1C,D) and is very essential to classify this Cu@Zinc oxalate nanomaterial as a heterostructured nanobrass alike the Cu@ZnO type. For BM1, the estimated structure and composition are given as α-Cu_40_Zn_25_/γ-Cu_11_Zn_24_ with maximum zinc contents of 38 and 68% in the α-brass and γ-brass phases, respectively. Crystallite size was calculated at 33 nm and is very close to the TEM size (40–45 nm).

**Figure 3:**
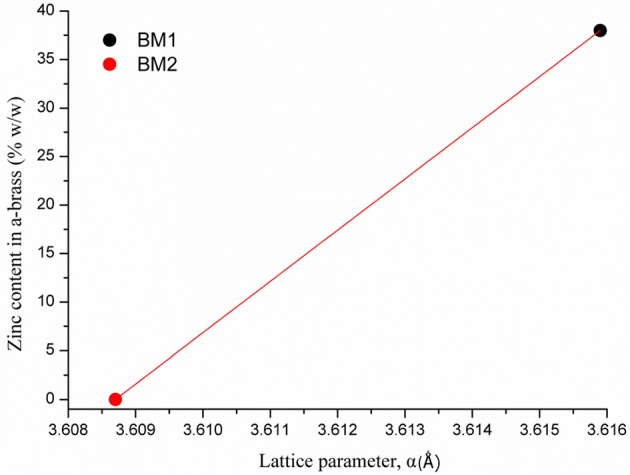
Linear fitting of zinc content in α-brass vs. lattice parameter. BM1 and BM2 represent the two boundaries of the fitting, maximum Zn in α-brass and monometallic copper, respectively.

As crystallite size is mainly governed by peak area and width, the 2θ of the peak summit can be correlated with the composition of the BMNPs that slightly alters the unit cell (Antonoglou et al., 2017, 2018; Zhou et al., 2018). Moreover, during atomic substitution in the wet chemical alloying process of nanobrass, the relatively bigger size of zinc atoms enlarges the lattice parameter when metallic copper turns into α-brass, as illustrated in Figure 3. According to this diagram displaying the linear fitting of zinc content in α-brass, the lattice parameter was calculated at 3.6159 Å (0.0028) and 3.6087 Å (0.0018) for the α-Cu_40_Zn_25_ (BM1) and Cu (BM2), respectively. As the BM1 and BM2 represent the two boundaries of the fitting (maximum Zn in α-brass and monometallic copper, respectively), it can further be very useful as a reference diagram for the estimation of α-brass zinc content in multifaceted brass materials with ZnO phases in the shell of the core nanocrystals.

### Conventional or Microwave Heating

**Figure 4:**
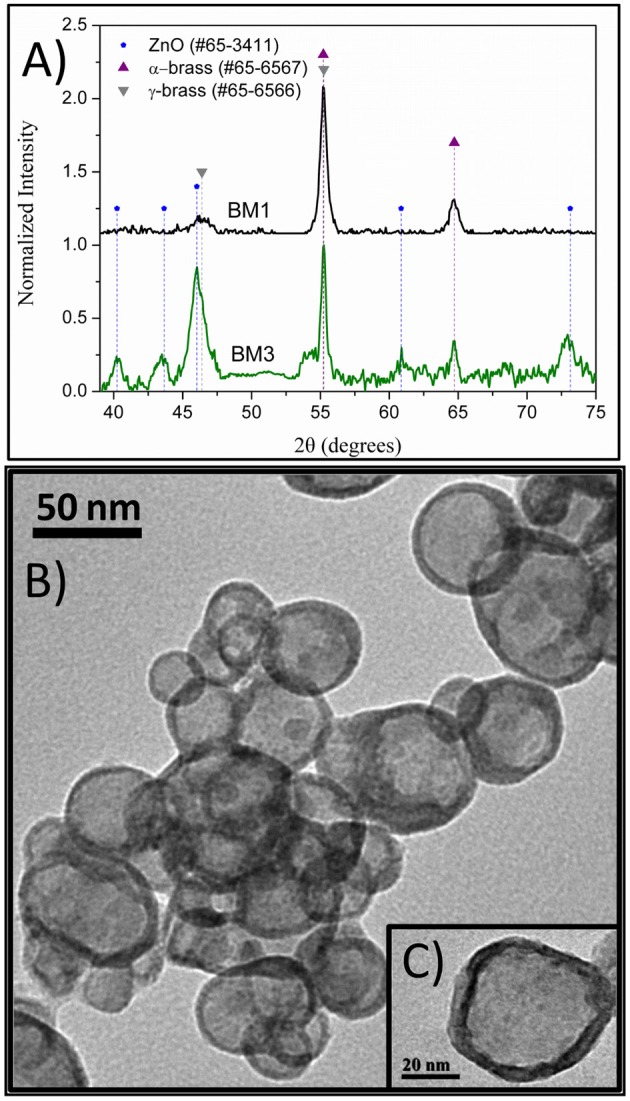
X-ray diffraction (XRD) patterns of BM1 (microwave) and BM3 (solvothermal) **(A)**. Transmition electron microscopy (TEM) images of α-Cu_59_Zn_30_@(ZnO)_11_ **(B,C)**.

To find out if conventional heating instead of microwave radiation can affect the composition and structure of the produced brass BMNPs, the same self-assembly reaction was tested through a solvothermal approach, where extended nucleation and growth steps take place. The polyol process solvothermal analog of a 30-min microwave polyol synthesis is considered at 6–8 h. Sample BM3 was fabricated by means of an 8-h solvothermal route at 240°C in the sole presence of TrEG. The composition of BM3 was estimated via SEM/EDX analysis (Figure S2) at Cu_59_Zn_41_ and is more copper-oriented than the 1:1 precursor ratio, still not very distant. Figure 4A depicts the XRD diffractograms where the ZnO phase emerged during the solvothermal synthesis (BM3). We assume that the zinc-rich γ-brass transformed to ZnO due to the extended nucleation and growth steps of solvothermal route that favors the most thermodynamically stable products and hydrolysis reactions. Regarding the α-brass zinc content of BM3, it was estimated via Figure 3 linear fit at 34% w/w based on the calculated lattice parameter of 3.6151 Å (0.0016), with an hierarchical overall structure of α-Cu_59_Zn_30_@(ZnO)_11_. The ZnO is deposited as a shell around the α-brass core, as shown in the TEM images (Figures 4B,C). Crystallite sizes were calculated as 35 nm for the α-Cu_59_Zn_30_ core and 13 nm for the ZnO shell, whereas TEM sizes were estimated at 45–50 nm.

### Effect of Precursor Ratio

**Figure 5:**
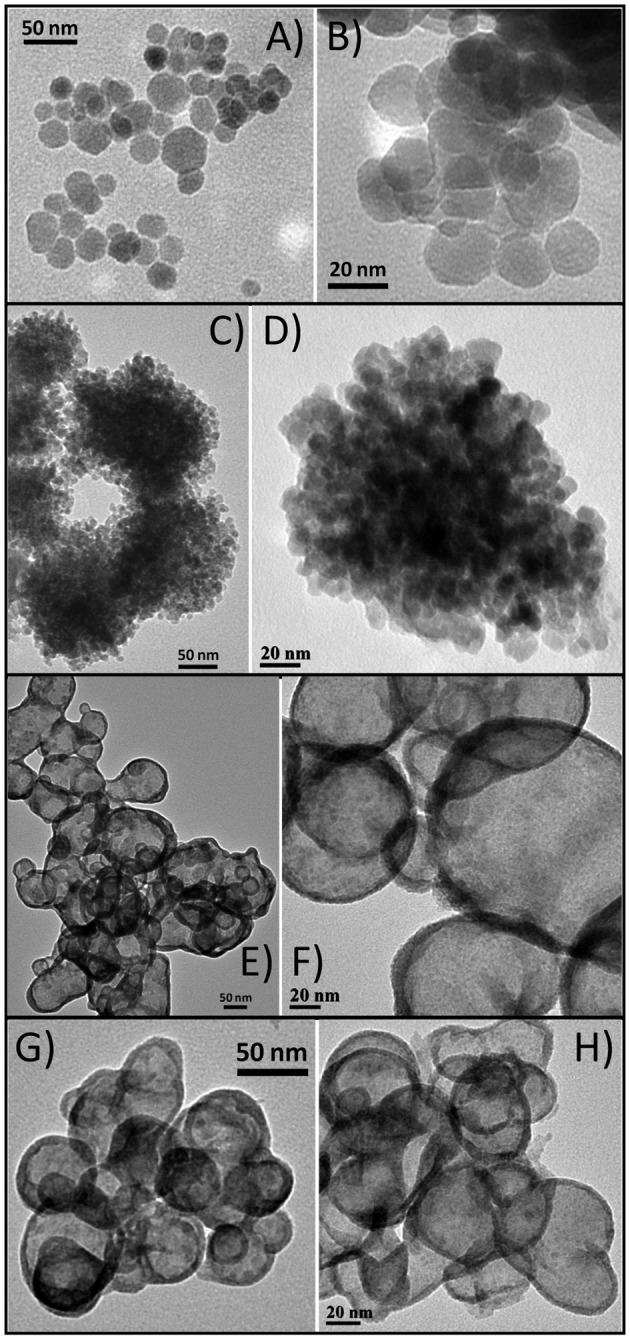
Transmition electron microscopy (TEM) captions of α-Cu_63_Zn_37_ **(A,B)**, α-Cu_47_Zn_10_/γ-Cu_19_Zn_24_ **(C,D)**, α-Cu_35_Zn_16_@(ZnO)_49_ **(E,F)**, and α-Cu_37_Zn_18_@(ZnO)_45_ **(G,H)**.

As the structure/composition of brass BMNPs is greatly affected by the content of the two metals, different precursor ratios were investigated during the synthesis. Four samples were prepared using solvothermal and microwave routes at 2:1 copper:zinc precursor ratio, BM4 and BM5, respectively, and at 1:2 copper:zinc precursor ratio, BM6 and BM7, respectively. Morphological characteristics of BM4, BM5, BM6, and BM7 were examined by TEM, and features (two captions for each sample) are given in Figure 5. Characteristic morphological traits appeared, namely, the core/shell hierarchy was observed for BM6 and BM7. Particle sizes were estimated at 25–30, 20–25, 55–65, and 45–50 nm for BM4, BM5, BM6, and BM7, respectively.

**Figure 6:**
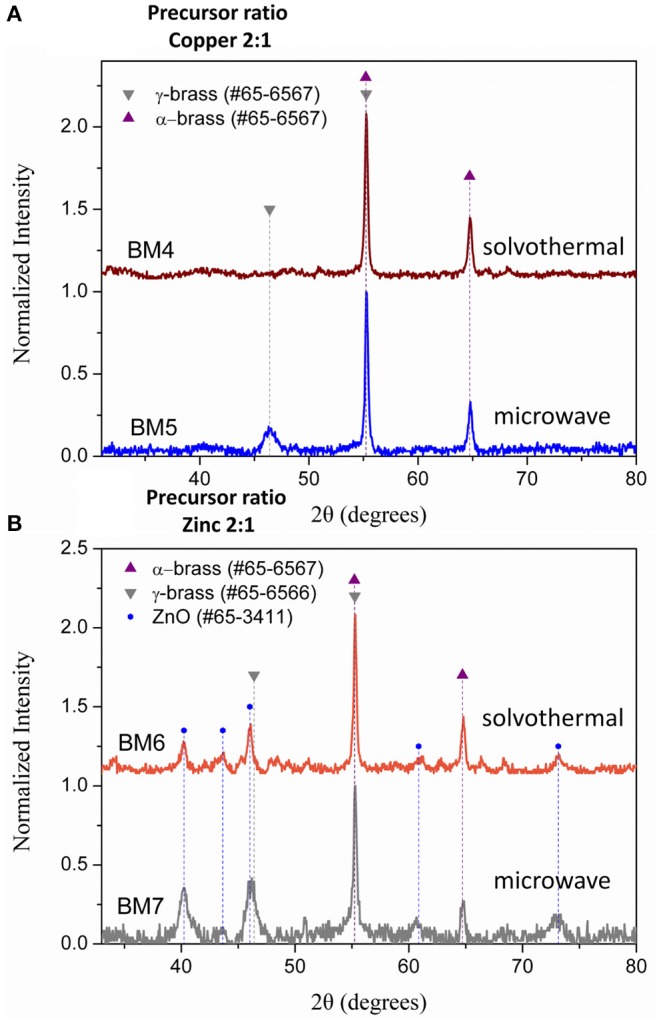
X-ray diffraction (XRD) patterns of BM4 (2:1 copper:zinc precursor ratio, solvothermal), BM5 (2:1 copper:zinc precursor ratio, microwave) **(A)**, BM6 (1:2 copper:zinc precursor ratio, solvothermal), and BM7 (1:2 copper:zinc precursor ratio, microwave**) (B)**.

Composition derived by SEM/EDX analysis (Figure S3) is given as Cu_63_Zn_37_, Cu_66_Zn_34_, Cu_35_Zn_65_, and Cu_37_Zn_63_ for BM4, BM5, BM6, and BM7, respectively, revealing a close match to the initial precursors' ratios. Figure 6 illustrates the XRD patterns recorded for BM4, BM5 (Figure 6A), BM6, and BM7 (Figure 6B). In case of copper precursor excess (BM4 and BM5), both samples were crystallized as pure BMNPs. Sample BM4, prepared through solvothermal route, adapted the α-brass structure, whereas BM5 fabricated via the microwave-assisted way displayed both α-brass and γ-brass phases. Although the initial zinc content was lower in both samples compared to copper, interestingly, the γ-brass was still observed in BM5 that was produced by the microwave synthesis. This indicates that the γ-brass is formed under microwave irradiation even when the α-brass is unsaturated while when extended nucleation and growth steps used (solvothermal approach), it is transformed into α-brass as a final point. The estimated composition/structure of BM4 is given as α-Cu_63_Zn_37_ with 37% w/w zinc content in the α-brass. By utilizing the quantitative option of MDI's Jade, the % wt crystal composition for BM5 is calculated at 57% α-brass and 43% γ-brass and an estimated composition/structure of α-Cu_47_Zn_10_/γ-Cu_19_Zn_24_ with 18 and 68% w/w zinc content in the α-brass and γ-brass, respectively. These values (37 and 18% w/w zinc content in the α-brass) are very close to the theoretical magnitudes of 35% and 21% zinc content in α-brass estimated via Figure 3 linear fit for BM4 and BM5, respectively. Lattice parameters were estimated at 3.6153 Å (0.0017) and 3.6130 Å (0.0017) for BM4 and BM5, respectively.

**Figure 7:**
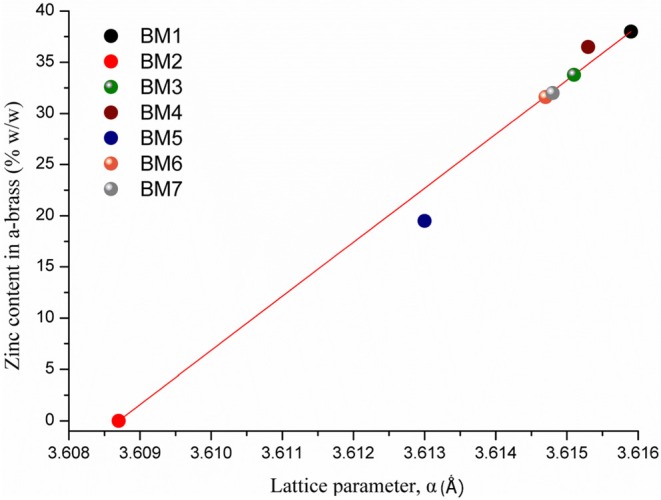
Linear fitting of zinc content in α-brass vs. lattice parameter for all as-produced nanoparticles (NPs).

Samples prepared under zinc precursor excess (BM6 and BM7) crystallized as hierarchical core-shell nanoarchitectures of α-brass@ZnO alike sample BM3. The Figure 3 linear fit was applied and gave 31 and 32% zinc content in the α-brass for BM6 and BM7, respectively with an estimated composition/structure of α-Cu_35_Zn_16_@(ZnO)_49_ and α-Cu_37_Zn_18_@(ZnO)_45_, respectively. Lattice parameters were estimated at 3.6147 Å (0.0024) and 3.6148 Å (0.0013) for BM6 and BM7, respectively. This is expected to some extent because an excess of zinc during the synthesis will lead to the most stable phase of ZnO via hydrolysis reactions after partially doping the α-brass. Crystallite sizes were calculated at 34, 35, 33/20, and 30/12 nm for α-Cu_63_Zn_37_ (BM4), α-Cu_47_Zn_10_/γ-Cu_19_Zn_24_ (BM5), Cu_35_Zn_16_@(ZnO)_49_ (BM6), and α-Cu_37_Zn_18_@(ZnO)_45_ (BM7), respectively. Figure 7 depicts the overall chart of the linear fitting of zinc content in α-brass vs. the calculated lattice parameter of all as-produced NPs, whereas Table 2 illustrates the lattice parameter values. It is clearly demonstrated that, with increasing zinc content in the α-brass, the lattice parameter increases.

**Table 2:** Zinc content in α-brass in correlation with estimated lattice parameter.

**Sample**	**Zinc content in α-brass** **(%)**	**Lattice parameter** **(Å)**
BM1	38	3.6159 (0.0028)
BM4	37	3.6153 (0.0017)
BM3	34	3.6151 (0.0016)
BM7	32	3.6148 (0.0013)
BM6	31	3.6147 (0.0024)
BM5	18	3.6130 (0.0017)
BM2	0	3.6087 (0.0018)

### State of the Organic Coating

**Figure 8:**
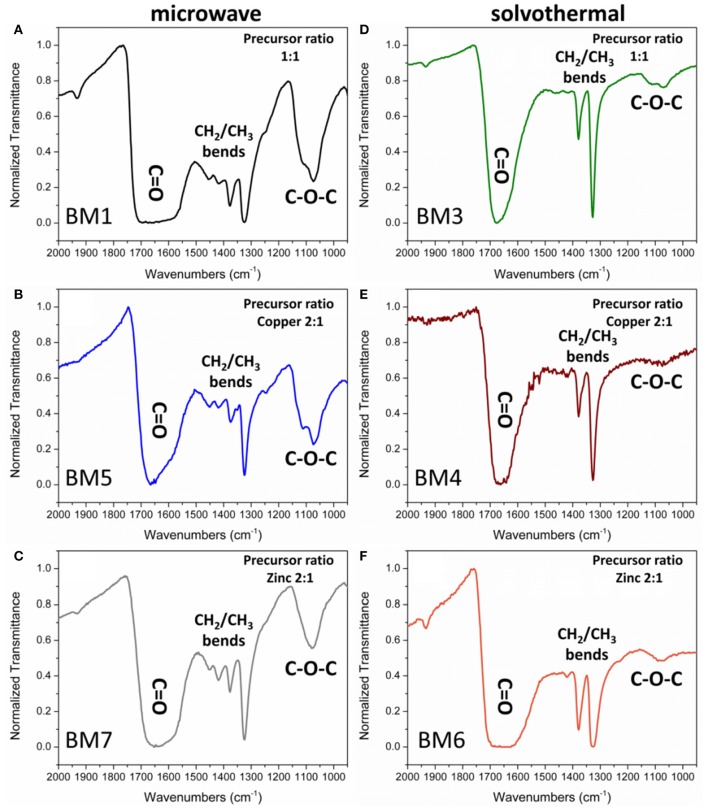
Fourier-transform infrared (FTIR) spectra of the organic coating of bimetallic nanoparticles (BMNPs) synthesized by microwave irradiation (BM1, BM5, BM7) **(A–C)** and solvothermal route (BM3, BM4, BM6) **(D–F)**.

The existence of the organic coating on the surface of the NPs has been confirmed by the FTIR spectra of the samples (Figure 8). The common nature of the polyols that have been used to functionalize the NPs surface is indicative in all cases while TrEG oxidation and fragmentation (Scheme 1) were inspected (Figure 8 and Figure S4).

Peaks of interest are examined in the 2.000–900 cm^−1^ region where ν-C = O (1.700–1.600 cm^−1^), ν-CH_2_ (1.500–1.400 cm^−1^), ν-CH_3_ (1.400–1.300 cm^−1^), and ν- C-O-C (1.200–1.000 cm^−1^) bonds are reflected. All samples displayed a broad large peak at 1.700–1.600 cm^−1^ region that is attributed to the plethora of oxidized forms of TrEG coming from one-end and/or two-end oxidized forms, such as aldehydic, ketonic, carboxylic, oxalate groups on the surface of BMNPs (Caizer and Stefănescu, 2002; Stefănescu et al., 2007; Dong et al., 2015b; Vamvakidis et al., 2015; Antonoglou et al., 2017; Tryfon et al., 2019). Broad peaks indicate that NPs can simultaneously be functionalized with more than one type of ligand. Furthermore, the ν-CH_3_ bends (1.400–1.300 cm^−1^) appeared dominant in all BMNPs. However, ν-CH_2_ (1.500–1.400 cm^−1^) and ν-C-O-C (1.200–1.000 cm^−1^) bends were significantly weak fainted for samples synthesized by the solvothermal route, revealing extensive fragmentation for the TrEG molecules and in specific the ether-type bonds of TrEG. Further evidence of the organic transformations is indicated from BM2 samples where oxalate forms were produced after the polyol fragmentation and finally Cu@Zinc oxalate BMNPs were synthesized (Figure S4).

**Figure 9:**
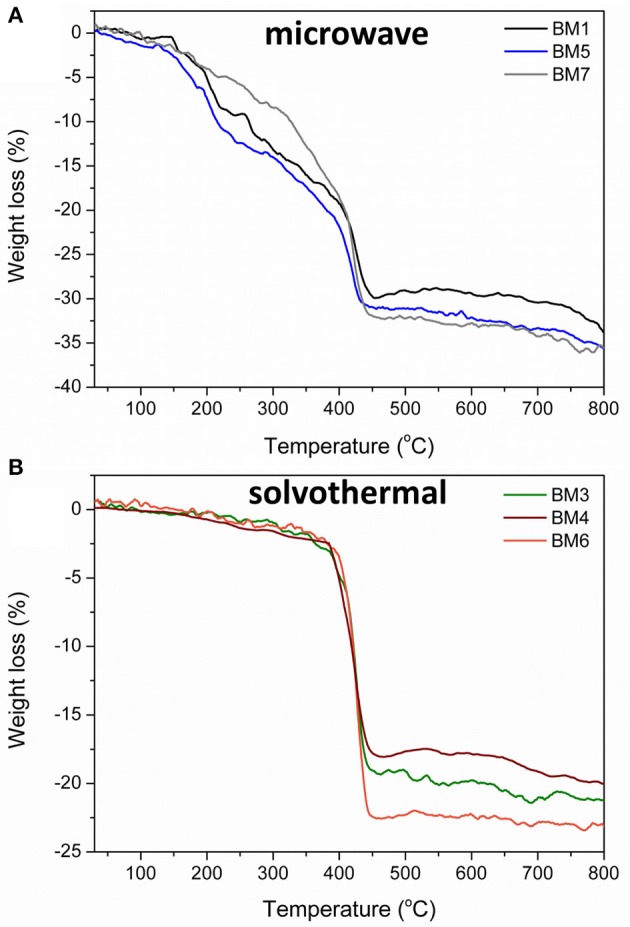
Thermogravimetric plots recorded during thermal treatment up to 800°C for BMNPs synthesized by microwave irradiation (BM1, BM5, BM7) **(A)** and solvothermal route (BM3, BM4, BM6) **(B)**.

The % w/w percentage of organic coating was measured by means of TGA. Figure 9 depicts the weight loss recorded for all BMNPs during thermal treatment up to 800°C, attributed to the decomposition of the surface organic layer. For BMNPs synthesized by microwave (BM1, BM5, BM7) 30–32% w/w organic coating was recorded, whereas for the products of the solvothermal route (BM3, BM4, BM6), 18–23% w/w. Moreover, multiple decomposition steps were observed for the microwave BMNPs that started from 150°C and unveiled a wide range of different forms of TrEG as coating. Also, the multiple decomposition steps and the relative higher % w/w organic content in case of microwave samples' BM1, BM5, and BM7 can be correlated to the polyalcoholic nature that gave rise to a “curl” type of coating around the BMNPs (Vamvakidis et al., 2015; Antonoglou et al., 2017), and this is in agreement with the displayed strong ν-C-O-C ether bonds (Figure 8). In contrast, a single decomposition step was recorded for the solvothermally prepared BMNPs that started right after 350°C, and in this case, a more thermally stable coating was established. Additionally, the fragmentation of polyol that occurred led to shorter alcohol chains that are unable to “curl” around the BMNPs and gave lower % w/w organic content.

### Aqueous Colloidal Stability

The colloidal stability of aqueous suspensions of BMNPs (pH = 7) was evaluated with DLS and ζ-potential measurements to provide the hydrodynamic size and surface charge. Results are summarized in Table 3. Overall, values are very close among all BMNPs with DLS sizes of 140–260 nm. In all cases, ζ-potential values were found negative but are not exceeding the −30 mV threshold that is referred to as electrostatic stabilization of NPs, and thus, it is suggested that brass BMNPs are stabilized by steric repulsion forces. More negative values, higher than −10 mV that were displayed for BM3, BM4, and BM6 can be correlated with the presence of fragmented species of TrEG. Different coating mechanisms revealed the “curl” type in the case of non-fragmentation (BM1, BM5, BM7) vs. the fragmented one (BM3, BM4, BM6) that leads to more electronegative groups (carbonyl/carboxyl/hydroxyl) at the outer surface of the BMNPs.

**Table 3:** Structure, organic coating state, hydrodynamic size, and ζ-potential of bimetallic nanoparticles (BMNPs).

**Sample**	**Structure**	**Organic coating** **(% w/w)**	**Fragmentation of polyol**	**Hydrodynamic size** **(nm)**	**ζ-potential** **(mV)**
BM1	α-Cu_40_Zn_25_/γ-Cu_11_Zn_24_	30	No	139	−5.4
BM3	α-Cu_59_Zn_30_@(ZnO)_11_	19	Yes	161	−10.5
BM4	α-Cu_63_Zn_37_	18	Yes	255	−12.6
BM5	α-Cu_47_Zn_10_/γ-Cu_19_Zn_24_	31	No	176	−7
BM6	Cu_35_Zn_16_@(ZnO)_49_	23	Yes	145	−13
BM7	α-Cu_37_Zn_18_@(ZnO)_45_	32	No	202	−6.1

## Conclusions

The physicochemical properties of BMNPs can be modulated widely and precisely, providing wonderful opportunities to improve their performance for many different technological applications. At the present study, getting advantage of our know-how on the use of the polyol toolbox was proven fruitful and revealed important aspects of the formation mechanism of brass NPs. The polyol process proved effective in fabricating different structures of hydrophilic nano-brass, namely, both bimetallic (CuZn) and bimetallic-oxide hierarchical core-shell (CuZn@ZnO) NPs with a variety of compositions ranging from copper-rich to zinc-rich materials. Moreover, estimated crystallite, particle, and hydrodynamic sizes were all very close, and thus, controlled regulation of only compositions, morphology, structure, and ZnO shell thickness was achieved. In that manner, a hydrophilic brass NPs' collection is accumulated, and the diverse characteristics of these NPs can be evaluated in antimicrobial, biomedical, and agrochemical applications. We have already initiated the endeavor with phytotoxicity evaluation and antifungal activity (Antonoglou et al., 2018) and aspire to unveil the true potential of hydrophilic nano-brass with future experiments and syntheses.

## Data Availability Statement

All datasets generated for this study are included in the article/Supplementary Material.

## Author Contributions

CD-S: conceptualization and supervision. CD-S, OA, and EF: methodology. OA, EF, VK, EP, and GL: validation. OA and EF: formal analysis and writing—original draft preparation. OA, EF, and VK: investigation. CD-S, NT, EP, GL, and SM: resources. OA and SM: data curation. CD-S, NT, SM, and GL: writing—review and editing. CD-S, NT, and SM: project administration and funding acquisition.

### Conflict of Interest

The authors declare that the research was conducted in the absence of any commercial or financial relationships that could be construed as a potential conflict of interest.
