# Supplementary material for: Structure Differentiation of Hydrophilic Brass Nanoparticles Using a Polyol Toolbox
Source: Front Chem. 2019 Nov 29;7:817. doi: 10.3389/fchem.2019.00817 (PMC6897281; doi:10.3389/fchem.2019.00817)
Supplement: Supplementary file 1 [file Table_1.DOCX]

**Figure S1**. SEM images of BM1 and BM2.

**Figure S2**. SEM image of BM3.

**Figure S3**. SEM images of BM4, BM5, BM6 and BM7.





**Figure S4**. FTIR spectra of the organic coating of BM2.
